# Prevalence of protein, lysine, and tryptophan inadequacy and their relation to stunting among Malawian under five children: evidence from the 2019/20 Integrated Household Survey

**DOI:** 10.3389/fnut.2026.1744220

**Published:** 2026-03-18

**Authors:** Keston Mwiwa Sinkhonde, Innocent Pangapanga-Phiri, Gareth Osman, Alexander A. Kalimbira

**Affiliations:** 1 Department of Human Nutrition and Health, Bunda College, Lilongwe University of Agriculture and Natural Resources, Lilongwe, Malawi; 2 Centre for Agricultural Research and Development, Bunda College, Lilongwe University of Agriculture and Natural Resources, Lilongwe, Malawi

**Keywords:** amino acids, child stunting, inadequacy, lysine, protein, tryptophan, Malawi

## Abstract

**Background:**

Despite Malawi facing a high burden of multiple nutrient deficiencies arising mostly from low dietary supplies, nutrition assessments focus largely on micronutrients (e.g., minerals and vitamins), ignoring macronutrients such as protein. This study aimed to assess prevalence of inadequate dietary intake of protein and two essential amino acids lysine and tryptophan and their relation to stunting among Malawian under five children.

**Methods:**

We extracted data on household food consumption, expenditure and anthropometry for 5,952 children aged 6–59 months from the 2019/20 Malawi’s Fifth Integrated Household Survey. Household food consumption and expenditure data were processed in R Studio with outliers replaced using median values, edible portions derived using conversion factors and all data standardized using Adult Female Equivalent (AFE). Relevant Food Composition Tables and database were utilized to estimate apparent intake of total and available protein, lysine, and tryptophan. Nutrient adequacy was assessed using the cut-off method based on Estimated Average Requirements, applying FAO thresholds. Anthropometric data were used to assess stunting, calculated using Z-scores in accordance with the 2006 WHO Growth Standards.

**Results:**

Respectively, inadequate available protein, lysine and tryptophan were prevalent in 49.0%, 56.4% and 25.7% of the children. Stunted children faced worse deficits – 52.5% inadequate available protein vs. 47.2% in non-stunted children (*p* < 0.001). Inadequate available lysine intake (61.5%) was most evident in the Central region, available protein inadequacy was higher in rural (50.7%) than urban areas (39.2%) (*p* < 0.001), whereas available tryptophan gaps were significantly greater (*p* = 0.009) in female- (28.7%) than male-headed households (24.6%).

**Conclusion:**

Inadequate intake of protein, lysine, and tryptophan was prevalent among children, particularly among those who were stunted and those living in rural areas. Regional variations were evident, with lysine inadequacy most pronounced in the Central region, while tryptophan inadequacy was more prevalent in female-headed households.

## 1 Introduction

Child malnutrition, particularly stunting, remains a major public health concern in Malawi, driven in part by widespread nutrient inadequacy, despite national commitments and efforts to curb the issue. By 2024, 38% of children under the age five were stunted (i.e., short for their age) (1), which is an indicator of chronic undernutrition. Although this represents a decline from 55% in 1992 (1), prevalence remains alarmingly high based on global thresholds for stunting (2). Children who experience stunting early in life face long-term consequences that continue into adulthood, including poor performance in school, and they earn less as adults (3). In Malawi, only 24% of children under five meet the minimum dietary diversity (MDD) threshold, consuming at least five out of eight defined food groups, highlighting inadequate nutrient intake and a major contributor to child malnutrition (4). Emerging evidence shows a high risk of nutrient inadequacy, particularly in protein and essential amino acids (EAAs) such as lysine and tryptophan in Malawian households, aggravated by the poor quality and limited availability of dietary protein sources (5).

Proteins are organic compounds comprising hydrogen, carbon, nitrogen, oxygen, and sometimes sulfur atoms, linked in a chain of amino acids (6). They play vital roles in the body, including supporting growth, repairing worn out tissues, facilitating metabolic processes, and regulating the balance of fluids (6). The quality of protein source is determined by complete availability and diversity of all EAAs, assessed in comparison to a reference protein that represents the standard of nutritional value (7). Adequate dietary intake is determined by estimated average requirement of 0.66 g/kg body weight per day for protein, 35 mg/kg for lysine, and 4.8 mg/kg for tryptophan; dietary intake below these levels is considered nutritionally inadequate (7).

Lysine is an essential amino acid required for protein synthesis, transportation of long-chain fatty acids into mitochondria for energy production, and maintenance of healthy bones by minimizing calcium excretion (8, 9). Together with tryptophan, the two are the most limiting EAAs in plant-based diets, particularly in cereals and grains such as maize, wheat, and rice, where their concentrations are often insufficient to meet human requirements (10). In addition to being a building block of protein, tryptophan is necessary for two key biological functions, which are synthesis of the neurotransmitter serotonin that regulates appetite, mood and sleep (11) and the production of metabolites involved in the biosynthetic of nicotinamide adenine dinucleotide (NAD or NADH), a coenzyme essential for cellular energy metabolism (12). Tryptophan is also metabolized into serotonin, a neurotransmitter that modulates the brain-gut axis, influencing appetite regulation (12). Inadequate dietary intake of tryptophan results into reduced serotonin synthesis, which has been associated with diminished appetite and lower food intake (13).

Adequate intake of protein and amino acids is positively associated with linear growth, underscoring the need for interventions that promote increased consumption of high protein local foods for optimal dietary intake among children (14). In Malawi, however, there is limited supply of protein, lysine and tryptophan, particularly among the poorest households due to diets that are predominantly cereal-based and deficient in essential amino acids (5). Inadequate intake of protein and essential amino acids may be a contributing factor to the persistently high prevalence of stunting among children in Malawi. Despite growing concern, empirical evidence linking specific amino acid deficiencies to child stunting in Malawi remains limited. Most national nutrition assessments focus on energy and general protein intake, overlooking the role of amino acid composition in growth outcomes. Therefore, this study aimed to address this gap by estimating the prevalence of protein, lysine, and tryptophan inadequacy and examining their relationships with stunting among Malawi’s under five children aged 6–59 months, using nationally representative data from the 2019/20 Integrated Household Survey.

## 2 Materials and methods

### 2.1 Food consumption data

We utilized data from Malawi’s Fifth Integrated Household Survey (IHS5), conducted nationwide between April 2019 and April 2020. The IHS is a World Bank-supported Living Standards Measurement Study (LSMS), implemented every 3–7 years in collaboration with the Malawi National Statistical Office (NSO) to collect nationally representative data on household living conditions, including poverty, income, health, education, and access to basic services (15).

Malawi’s Fifth Integrated Household Survey was a cross-sectional study that used a stratified two-stage sampling design. The primary sampling units (enumeration areas) were selected at the initial sampling stage. An enumeration area (EA) is the smallest area with defined boundaries established for the purpose of national census with an approximate of 235 households in each (15). A total of 11,434 households from the 750 EAs were interviewed out of 12,000 sampled households because of coronavirus (COVID-19) pandemic during the survey implementation period (15).

The dataset was retrieved from the World Bank website https://microdata.worldbank.org/index.php/catalog/3818 following written permission from the Malawi National Statistical Office (NSO) on December 18, 2024. For this study, children under the age of 5 years formed the study population, hence a sub-sample of 5,952 children aged 6–59 months was drawn from the 11,434 households. This sub-sample drew children who were aged 6–59 months and had plausible anthropometric data based on stature (length/height) and age to compute stunting using thresholds set by World Health Organization (WHO) (2). Data extracted from the IHS5 included information on food consumption, anthropometry, household demographic characteristics, and survey weights. Household food consumption data was collected using a predefined list of 135 food items with a 7-days recall period. Specifically, respondents were asked: “Over the past 7 days, did you or others in your household consume any [item]? How much in total did your household consume in the past 7 days?”

#### 2.1.1 Food matching

The food composition table (FCT) used in this study was adapted from the dataset developed for the 2016/17 Malawi Fourth Integrated Household Survey (IHS4), as described by Muleya (16). This adaptation was appropriate because the food item list in IHS4 and the 2019/20 IHS5 are largely comparable. Nutrient values were compiled for both total and available protein, as well as for the indispensable amino acids, including lysine and tryptophan. In this context, “total protein” refers to the complete protein content of a food item, while “available protein” reflects the fraction that is digestible and bioavailable for human metabolism (7). In brief, approximately 72% of the nutrient values were sourced from the Malawi FCT (17), with an additional 26% drawn from the South Africa FCT (18), primarily to account for processed foods such as biscuits, infant cereals, formulas, and beverages that were not represented in the Malawi database. A small proportion (less than 3%) of food items were matched using data from the West Africa FCT (19) and the USDA FoodData Central Database (20). Due to limited regional data on amino acid composition, lysine and tryptophan values were predominantly obtained from the USDA FoodData Central Database – which is consistent with a study by Muleya et al. (5).

#### 2.1.2 Calculation of adult female equivalent

We estimated apparent household food consumption and calculated daily intakes of protein, lysine, and tryptophan in grams per adult female equivalent (AFE). The AFE metric assumes that food within a household is distributed proportionally to the energy requirements of each member, standardized to a reference individual: a non-pregnant, non-lactating woman aged 18–29 years. This reference individual represents one AFE (21). The metric, adopted from a study by Tang et al. (21) was used because it reflects the average energy and nutrient intake required to meet the needs of most household members (21). To compute AFE values, we followed the methodology described by Tang et al. (21). Briefly, energy requirements for each household member were estimated based on age, sex, physiological status (e.g., pregnancy or lactation), and average body weight, using guidelines from the Joint FAO/WHO/UNU Expert Consultation on Human Energy Requirements (22). We applied a moderate Physical Activity Level (PAL) of 1.6 × basal metabolic rate (BMR) for all adults, consistent with the approach described by Tang et al. (21). This value was chosen to reflect the typical energy-expenditure patterns observed in the general Malawian population (16). For women of reproductive age, an average body weight of 55 kg was applied, based on data from the 2015/16 Malawi Demographic and Health Survey (23). For breastfed children, we estimated energy intake from complementary foods by subtracting the age-specific energy contribution of breastmilk from their total daily energy requirement (24). Total energy intake from breast milk was estimated using values reported in a WHO (24) systematic review of studies conducted in developing countries, stratified by infant age groups (0–2, 3–5, 6–8, 9–11, and 12–23 months). For infants aged 3–23 months, average energy contributions from breast milk were 434 kcal (3–5 months), 413 kcal (6–8 months), 379 kcal (9–11 months), and 346 kcal (12–23 months). Infants aged 0–2 months were assumed to be exclusively breastfed (25). Each household member’s AFE was then calculated by dividing their individual energy requirement by that of the reference female. The total household AFE was obtained by summing the AFE values of all members.

### 2.2 Assessment of nutrient adequacy

Estimated intakes of protein, lysine, and tryptophan were compared against the estimated average requirements (EARs) for a reference adult female; non-pregnant, non-lactating, aged 18–29 years. The EARs used were: 36.3 g/day for protein, 1.95 g/day for lysine, and 0.26 g/day for tryptophan. The EAR represents the daily intake level sufficient to meet the nutritional needs of 50% of individuals in a specific age- and sex-defined population group (5). These EAR values correspond to FAO/WHO/UNU (7) recommendations of 0.66 g/kg/day for protein, 35 mg/kg/day for lysine, and 4.8 mg/kg/day for tryptophan (7). Accordingly, any apparent intake below the respective EAR was classified as inadequate. The primary outcome variables of the study were the prevalence of inadequate intake of total and available protein, lysine, and tryptophan. These were analyzed across key demographic characteristics, including child nutritional status, geographic region, urban/rural residence, household headship, and caregiver literacy level.

### 2.3 Statistical analysis

#### 2.3.1 Preprocessing of household consumption data

Household consumption quantities in IHS5 were recorded in standard units and non-standard units. Therefore, we pre-processed food consumption data to convert consumption quantities in non-standard units (e.g., basin, pail and plate) into standard units (e.g., kilograms) which were further reduced from kilograms to grams by multiplying quantities in kilograms by 1000 g to derive consumption quantities in grams per household per week. Further, we converted weekly household consumption by divided by 7 to obtain daily household consumption.

Reported household food items were converted into edible portions using IHS5 conversion factors to better reflect actual consumption excluding inedible components like bones in meat and fish. We obtained these factors from the World Bank website https://microdata.worldbank.org/index.php/catalog/3818. We identified and managed outliers by applying a log transformation, which helped approximate a normal distribution (26). Observations exceeding +5 standard deviations were flagged as outliers and replaced with the median intake for the corresponding food item. In contrast, we retained lower outliers as they were considered plausible, reflect genuinely low intake within the context of the study (26).

Anthropometric data which contained height/length and age for children aged 6–59 were imported into WHO Anthro to generate height-for-age Z-scores. Height-for-age Z-score values below −6 standard deviations (SD) or exceeding +6 standard deviations (SD) were considered implausible (27), and were excluded from the dataset. The dataset of 5,952 children was exported to Microsoft Excel and subsequently merged with the main dataset using Stata/SE version 17.0 for Windows (64-bit x86-64), Revision 05 May 2021 (Stata Corp LLC, College Station, TX). Children who were <−2 SD were considered stunted while those equal ≥−2 SD were considered non-stunted (27).

#### 2.3.2 Data analysis

We used version 17.0 of Stata/SE for Windows (64-bit x86-64), Revision 05 May 2021 (Stata Corp LLC, College Station, TX) to summarize continuous variables into frequencies, means and standard errors. Pearson chi-square test was conducted to measure the association between predictor variables (e.g., nutrition status, region, residence, sex of household head, and caregiver literacy level) and outcome variables (e.g., prevalence of inadequate intake of total and available protein, lysine, and tryptophan). Statistical significance was set at *p* < 0.05, implying that findings were considered significant if the likelihood of occurring by chance was less than 5%. All estimates were weighted using the IHS5 survey sampling weights to ensure representativeness.

### 2.4 Ethical approval

The study protocol was reviewed by the Lilongwe University of Agriculture and Natural Resources Research Ethics Committee (LUANAR-REC), approval number 2025-0012 on 4 April, 2025.

## 3 Results

### 3.1 Characteristics of children and their parents

Table 1 presents the key characteristics of children and their parents summarized using frequencies and means with corresponding standard errors (SE). The analysis revealed that children had a mean age of 32.1 months (SE = 23.7), mean height of 85.8 cm (SE = 17.8), and a mean height-for-age Z-score of −1.5 (SE = 2.4), based on 5,952 participants. For parents, the average age of mothers was 32.7 years (SE = 17.0; *n* = 5,876) while the mean age of fathers was 36.7 years (SE = 20.0; *n* = 4,489).

**TABLE 1:** Characteristics of children and their parents.

Characteristic	Frequency	Mean ± SE
Age of child (months)	5,952	32.1 ± 23.7
Height of child (centimetres)	5,952	85.8 ± 17.8
“Height for age” (Z-scores)	5,952	−1.5 ± 2.4
Age of mother (years)	5,876	32.7 ± 17.0
Age of father (years)	4,489	36.7 ± 20.0

Values are means ± standard errors (SE).

Table 2 shows stunting prevalence by household and environmental characteristics. Analysis revealed that there was a relationship between stunting among children and various housing and environmental characteristics across the study population in Malawi. For instance, stunting prevalence was significantly higher among children from households with unimproved walls (20.1%) compared to those with improved walls (15.4%) (*p* < 0.001). Similarly, children from households with unimproved roofs had a higher stunting prevalence (19.5%) than those with improved roofs (16.0%) (*p* < 0.005). The presence of a hand washing facility was also associated with lower stunting rates; children from households without hand washing had a stunting prevalence of 33.5%, which was significantly higher than 1.9% among those with access to hand washing facility (*p* = 0.005). Additionally, stunting prevalence was significantly higher among children living in households not sleeping under bed nets 30.2% compared to children from households who were sleeping under bed nets 5.3% (*p* < 0.003). Similarly, stunting prevalence was higher among children from households without electricity (32.8%) compared to those with electricity (2.7%) (*p* < 0.017). In contrast, there was no statistically significant association between stunting and the presence of improved rubbish disposal and toilet facility although the frequency distribution indicated a notable difference, as detailed in Table 1 (*p* > 0.05).

**TABLE 2:** Stunting prevalence by household and environmental characteristics.

Characteristics	Category	Stunting prevalence (%)	Number of children (*n*)	*P*-value
Walls	Improved	15.4	910	0.001
	Unimproved	20.1	1,191	
Roofs	Improved	16.0	949	0.005
	Unimproved	19.5	1,156	
Dwelling with electricity	Yes	2.7	157	0.017
	No	32.8	1,943	
Rubbish disposal	Improved	13.6	806	0.231
	Unimproved	21.9	1,299	
Toilet	Improved	11.7	694	0.373
	Unimproved	23.7	1,405	
Hand washing facility	Available	1.9	113	0.005
	Unavailable	33.5	1,988	
Under five children sleeping under bed net	Yes	5.3	312	0.003
	No	30.2	1,789	

*P*-values were generated through chi-square test. Significance was tested at *p* < 0.05.

### 3.2 Prevalence of apparent nutrient inadequacy

#### 3.2.1 Overall prevalence of apparent nutrient inadequacy (total and available protein, lysine and tryptophan) among children under the age of five years in Malawi

**FIGURE 1:**
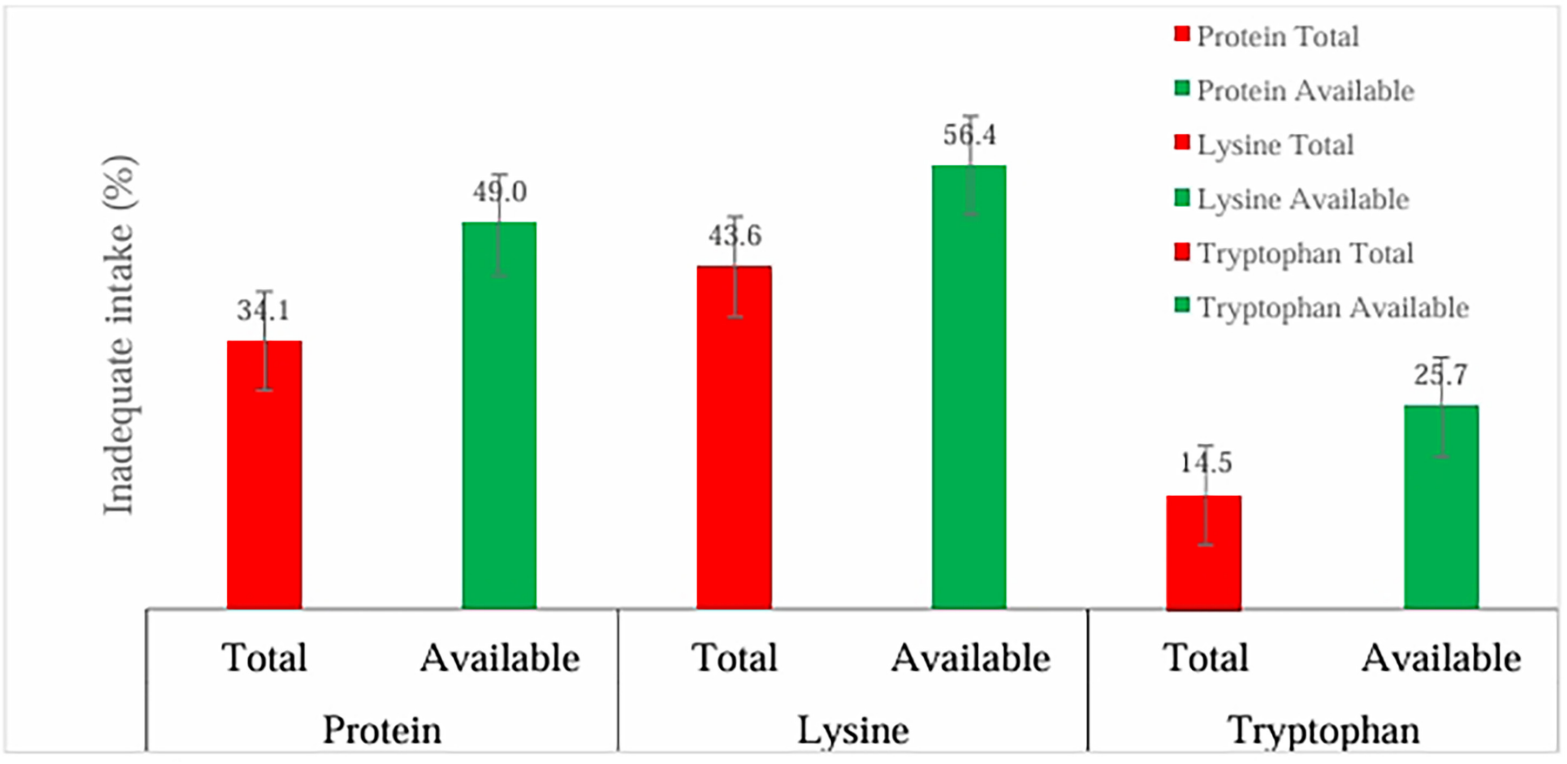
Overall prevalence of apparent inadequacy in total and available protein, lysine, and tryptophan (*n* = 5,952).

Figure 1 shows the overall prevalence of apparent nutrient inadequacy (total and available protein, lysine, and tryptophan) among Malawian children under 5 years (*n* = 5,952). While 34.1% of children had inadequate total protein intake, the proportion increased to 49.0% when considering available protein. A similar pattern was observed for lysine, with inadequacy rising from 43.6% for total intake to 56.4% for available intake. Tryptophan inadequacy was also more pronounced when availability was considered, increasing from 14.5% to 25.7%. These results shows that apparent inadequacy on nutrient intake among children is compounded by availability.

#### 3.2.2 Prevalence of apparent nutrient inadequacy (total protein, lysine and tryptophan) by social and demographic characteristics

There was a significantly higher prevalence of apparent inadequate intake of total protein (37.1% vs. 32.5%; *p* = 0.003), lysine (47.6% vs. 41.4%; *p* < 0.001), and tryptophan (17.2% vs. 13.1%; *p* < 0.001) among households with stunted children compared to those with non-stunted children (Table 3). Regional analysis revealed a consistently higher prevalence of inadequacy across total protein (39.5%), lysine (49.4%), and tryptophan (19.7%) intake among children in the Central region (Table 3). For apparent total protein, the prevalence of inadequacy was 30.8% in the North, 39.5% in the Central, and 29.7% in the South, underscoring the Central region’s elevated burden of nutrient inadequacy while the prevalence estimates in the North and South were numerically close. Overall, statistical analysis revealed significant variation across the three regions (*p* < 0.001).

**TABLE 3:** Prevalence of apparent inadequate intake of total protein, lysine and tryptophan among Malawian under five children (*n* = 5,952) by demographic characteristics.

Characteristics	Protein		Lysine		Tryptophan	
	Prevalence (%)	*P*-value	Prevalence (%)	*P*-value	Prevalence (%)	*P*-value
Nutrition status						
Non-stunted Children	32.5	0.003	41.4	0.001	13.1	0.001
Stunted children	37.1		47.6		17.2	
Region						
Northern region	30.8	0.001	37.2	0.001	7.8	0.001
Central region	39.5		49.4		19.7	
Southern region	29.7		39.5		11.3	
Residence						
Rural	35.5	0.001	45.8	0.001	15.8	0.001
Urban	25.8		30.2		7.0	
Sex of household head						
Male	33.8	0.499	43.3	0.585	13.3	0.001
Female	34.1		44.3		17.9	
Literacy of caregiver						
Able to read (yes)	35.3	0.398	42.2	0.256	15.6	0.725
Able to write (yes)	42.0	0.220	42.0	0.220	15.4	0.734

*P*-values were generated through chi-square. Significance was tested at *p* < 0.05.

In terms of residence, rural areas depicted higher prevalence of apparent inadequate total protein, lysine and tryptophan intake than urban areas (*p* < 0.001). By household headship, nutrient inadequacy was generally similar with no statistically significant different between female- and male-headed households for protein (34.1% vs. 33.8%, *p* > 0.05) and lysine (44.3% vs. 43.3%, *p* > 0.05). However, tryptophan inadequacy was significantly higher among female-headed households (17.4%) compared to male-headed households (13%), with a highly significant difference (*p* < 0.001). This suggest that apparent inadequacy of tryptophan intake may be more pronounced in female-headed households compared to other nutrients.

#### 3.2.3 Prevalence of apparent nutrient inadequacy (available protein, lysine and tryptophan) by social and demographic characteristics

While Table 3 reported inadequacy based on apparent total nutrient intake, Table 4 shows inadequacy after accounting for nutrient availability (protein, lysine, and tryptophan) by demographic characteristics of the study population, including nutritional status, region, residence, sex of the household head, and caregiver literacy. Key findings indicate a higher prevalence of inadequate available protein among households with stunted children (52.5%) compared to those with non-stunted children (47.2%) (*p* < 0.001). Similarly, inadequate lysine intake was more prevalent among households with stunted children (60.2%) than those with non-stunted children (54.5%) (*p* < 0.001). For tryptophan, the prevalence was also higher in households with stunted children (29.4%) compared to 23.6% in households with non-stunted children (*p* < 0.001).

**TABLE 4:** Prevalence of apparent inadequacy of available protein, lysine and tryptophan among Malawian under five children (*n* = 5,952) by demographic characteristics.

Characteristics	Protein		Lysine		Tryptophan	
	Prevalence (%)	*P*-value	Prevalence (%)	*P*-value	Prevalence (%)	*P*-value
Nutrition status						
Non-stunted children	47.2	0.001	54.5	0.001	23.6	0.001
Stunted children	52.5		60.2		29.4	
Region						
Northern region	45.6	0.001	47.8	0.001	19.1	0.001
Central region	53.2		61.5		31.5	
Southern region	45.8		53.8		21.8	
Residence						
Rural	50.7	0.001	45.8	0.001	27.8	0.001
Urban	39.2		30.2		13.4	
Sex of household head						
Male headed	49.3	0.542	56.7	0.604	24.6	0.009
Female headed	48.2		55.8		28.7	
Literacy of caregiver						
Able to read	48.7	0.708	55.3	0.423	25.7	0.929
Able to write	48.7	0.686	55.4	0.439	25.7	0.962

*P*-values were generated through chi-square. Significance was tested at *p* < 0.05.

After accounting for nutrient availability, regional analysis showed higher prevalence of inadequacy in available protein (53.2%), lysine (61.5%), and tryptophan (31.5%) intake among children, with statistically significant differences observed in the Central region (*p* < 0.001) (Table 4). Across all nutrients, a consistent regional pattern was observed, with the Central region showing the highest prevalence of inadequacy. Prevalence of apparent available protein inadequacy was 45.6% in the North, 53.2% in the Central, and 45.8% in the South. The Central region stood out with the highest risk of nutrient inadequacy, while the prevalence estimates displayed in the North and South were close in value. Overall, statistical analysis demonstrated significant differences across the three regions (*p* < 0.001) (Table 4). Regarding residence, a significantly higher prevalence of inadequate apparent intake was evident in rural compared to urban areas for available protein (50.7% vs. 39.2%, *p* < 0.001), lysine (45.8% vs. 30.2%, *p* < 0.001), and tryptophan (27.8% vs. 13.4%, *p* < 0.001) (Table 4). This suggest that children in the rural may be more at risk of apparent nutrient inadequacy compared to their urban counterparts.

There was also a statistically significant higher prevalence of inadequate available tryptophan intake among female-headed households (28.7%) compared to male-headed households (24.6%) (*p* < 0.001). However, no significant association was found between the sex of the household head and the prevalence of inadequate available protein or lysine supply (*p* > 0.05). Similarly, no association was observed between caregiver literacy and the prevalence of inadequate protein, lysine, or tryptophan intake (*p* > 0.05).

## 4 Discussion

### 4.1 Overall prevalence of apparent nutrient inadequacy (total and available protein, lysine and tryptophan) among children under the age of five years in Malawi

The study revealed that the prevalence of inadequate available protein, lysine, and tryptophan was higher than that of total protein, lysine and tryptophan. This disparity suggests that the quality of dietary protein, rather than just the quantity, plays a critical role in meeting essential amino acid requirements such as lysine and tryptophan. Maize based sources of protein that dominate Malawian diets (28), characterized by low lysine and tryptophan content and limited digestibility may likely contribute to the higher prevalence of inadequacy in available protein and essential amino acids (29). A previous study showed that Malawi experienced a limited supply of protein and essential amino acids particularly lysine, exacerbated by the dominance of plant-based protein sources in the diet (5). In Malawi, 67% of total protein consumption comes from cereals (28) of which maize contributes 33.3% (28).

In Indonesia, cereal based diets have been associated with increased inadequacy in protein and essential amino acid intake due to their low protein content, incomplete essential amino acid profiles, and the presence of anti-nutritional factors such as phytates, trypsin and protease inhibitor that impair nutrient absorption (30). Maize dominates Malawi’s total agricultural land, occupies about 50% of total planted area (31), is a key driver of food inflation (31), and is consumed by 97.6% of the population (32). Continued overreliance of ordinary maize varieties, which are low in protein content and limiting amino acids lysine and tryptophan may drive the protein and amino acid inadequacy observed in this study among children (Figure 1). However, since maize is engrained in the Malawian culture (31), breeding programmes that produce varieties rich in protein quantity and quality are needed to improve population health (33). Adoption of quality protein maize (QPM) has been largely unsatisfactory in much of the maize-dependent world (34), suggesting that breeding programmes need to integrate social aspects of food to understand, preserve and breed for desirable traits (34, 35). Such breeding programmes should also integrate nutrition education to make the improved varieties known and increase chances of adoption.

### 4.2 Prevalence of nutrient inadequacy (total and available protein, lysine and tryptophan) by social and demographic characteristics

We found a higher prevalence of inadequate apparent intake of total and available protein, lysine and tryptophan among households with stunted children compared to those with non-stunted children (Tables 3, 4). While this is not a causal association, inadequate protein intake is known to reduce longitudinal bone growth (36), such that children who experience inadequate intake of protein are four times more likely become stunted than those with adequate intake (14). A cohort study conducted in Bangladesh found that animal protein intake during the first 24 months of life was significantly associated with improved linear growth among children under 2 years of age (37). This may likely be due to their roles in stimulating insulin-like growth factor-1 (IGF-1) that participates in cell differentiation and growth (38). The dominance of cereals as a primary source of protein in Malawian diets (28) may restricts not only the quantity but also the quality of the protein consumed by the children, which may further contribute to low linear growth (14).

Predominant plant-based diets in low and middle-income countries have been associated with high rates of child stunting, due to their limited protein content and poor digestibility (10). A study in Indonesia showed that stunted children had significantly lower intake of essential amino acids particularly isoleucine, histidine, and methionine compared to their non-stunted counterparts (*p* < 0.05) (30). A study in Kenya found that consumption of eggs and milk on more than 3 days was significantly associated with increased monthly height gain among children 0.010 cm/month (95% CI: 0.002–0.019) for eggs and 0.008 cm/month (95% CI: 0.004–0.013) for milk (39). In Malawi, there is low supply of available protein and essential amino acids among rural Malawians because of the predominance of plant based protein in Malawian diets that have low quality protein and EAAs (5). For instance plant protein accounts for the majority (cereals contributing an average of 66% of the protein, legumes and nuts supply 15% while 4% comes from vegetables). On the contrary, 11% of the protein intake comes from animal products while the remaining proportion being supplied by other foods (5). Taken together, we argue that plant-based diets were likely more prevalent, and therefore predictive of disparities in the prevalence of inadequate apparent nutrient (total and available protein, lysine and tryptophan) intake among households with stunted children compared to their non-stunted counterparts.

Regional analysis showed a statistically significant association in nutrient inadequacy (*p* < 0.001) for both apparent total and available intake, with the magnitude of differences varying by nutrient (Tables 3, 4). For instance, the prevalence of inadequate apparent intake of available protein, lysine and tryptophan, was high across regions with the Central region exhibiting the highest prevalence (53.2% for protein, 61.5% for lysine and 31.5% for tryptophan) (Table 4). The magnitude and regional distribution of inadequacy differed across nutrients, particularly for apparent total and available protein (Tables 3, 4). For instance, Apparent total protein inadequacy was estimated at 30.8% in the North, 39.5% in the Center, and 29.7% in the South, underscoring the Central region’s elevated burden of total protein inadequacy, while the North and South showed prevalence estimates that were numerically close (Table 3). Similarly, apparent available protein inadequacy was estimated at 45.6% in the North, 53.2% in the Center, and 45.8% in the South, highlighting the Central region’s elevated risk of available protein inadequacy, while the prevalence estimates in the North and South were of numerically close magnitude. Overall, statistical analysis demonstrated significant differences of nutrient inadequacy across the three regions (*p* < 0.001) (Table 4). Available evidence in Malawi shows regional economic and child nutrition disparities. For instance, daily per capita consumption was estimated at MK739 in the Northern region, MK553 in the Central region, and MK600 in the Southern region, with the Central region recording the lowest level (40). Furthermore, attainment of the minimum acceptable diet (MAD) among children aged 6–23 months varied across regions. For instance, MAD was estimated at 51.1% in the North, 37.8% in the Central Region, and 44.6% in the South, again with the Central Region experiencing the lowest attainment (41).

Regarding residence, significantly higher prevalence of inadequate apparent intake of total and available protein, lysine and tryptophan were observed in rural areas compared to urban areas. Residential economic disparities may be associated with disparities in apparent nutrient inadequacy considering that rural residents experience higher rates of poverty compared to their urban counterparts (40), a condition that undermines livelihood security including access to nutrient dense foods and essential services (42). Malawi’s poverty report of 2020 showed that 56.6% of the rural population lived in poverty, compared to 19.2% in urban areas (40). This highlights the need for resident-specific policy interventions, such as integrating nutrition education into poverty reduction programmes, to improve economic access to quality protein-rich foods particularly animal source foods among rural households.

In terms of household headship, female headed households showed statistically significant higher prevalence of inadequate apparent tryptophan intake compared to male-headed households. A Malawian study revealed that unequal power disparities in access to and control over productive resources between men and women contribute to the high prevalence of malnutrition including stunting among children in the Central region (43). Established evidence reveals that female-headed households are disproportionately affected by nutrition insecurity compared to their male-headed counterparts due to limited access to production resources and lower income levels (44). For example, 56.8% of people in female-headed households were poor in 2019/2020 compared to 48.5% in male-headed households (40). It is also known that female-headed households occupy significantly smaller landholdings averaging 0.49 hectares compared to 0.61 hectares for male-headed households (45). Yet, despite this limited access to land, women contribute approximately 70% of household food production (31). It is known that women and girls experience persistent deprivations including food and nutrition (46). In Ethiopia and Nigeria, higher income levels and educational attainment were associated with increased food security among male-headed households compared to their female-headed counterparts (47) as these factors enhance both physical and economic access to food and contribute to its stability (48). In Brazil, food insecurity was associated with headship of the household where female headed households were more food insecure compared to their male headed counterparts (49). In Bangladesh, it was also evident that female headed households were less food secure compared to male headed counterparts (50). This highlights the urgent need for targeted policy interventions that promote diversification of food production within women’s farming systems to improve access to nutrient-dense foods. It also calls for institutional reforms to reassess agricultural land ownership policies through a gender-responsive lens. Further, assessing the effectiveness of income-focused interventions directed at female heads of household, such as employment support and expanded access to credit on household food security and child nutritional status would help reduce inadequate nutrient disparities among children.

There was no significant association between sex of the household head and the inadequacy of apparent intake of total and available protein and lysine, nor between caregiver literacy and the prevalence of inadequate total and available protein, lysine, or tryptophan supply (*p* > 0.05). A systematic review conducted for Sub-Saharan Africa showed conflicting findings among countries in the region on the association between education level of caregivers and prevalence of stunting among children (51). For example, in Uganda no statistically significant association was found between caregiver education level and prevalence of childhood stunting (*p* > 0.05), whereas in Cameroon, a positive correlation was observed where higher caregiver education was linked to lower rates of child stunting (51). Such conflicting results call for context-specific assessments and prioritization of interventions to improve food and nutrition security.

### 4.3 Study strengths and limitations

The findings from this study are uniquely relevant to the Malawian context, providing valuable insights to guide targeted nutrition programming and implementation aimed at sustainably improving child health and nutritional outcomes. Further, these findings may also serve as a foundation for prospective studies aimed at establishing causal relationships between inadequate nutrient intake and stunting, particularly in Malawi and other countries with similar socioeconomic and nutritional profiles. However, household consumption and expenditure surveys (HCES) like IHS5 have several limitations. As a cross-sectional survey, the IHS5 data cannot be used to draw causality, hence we do not claim that inadequate apparent intake of total and available protein, lysine and tryptophan was the cause of stunting in our findings. Additionally, the findings may be affected by under- or over-reporting, which could be intentional or not by respondents (52). Further, HCESs are associated with recall bias in which a respondent may forget foods consumed and their quantities in the context of low literacy and food insecurity (52). The AFE metric used, assumes that a non-pregnant, non-lactating woman represents the diverse consumption patterns and needs of every household members irrespective of variations in calorie and nutrient requirements for growth and development of under five children (53), and consumption quantities for children were estimated using this metric, although its validity remains uncertain (52). Additionally, the study did not examine sustainability aspects related to social and economic dimensions (e.g., household income, food affordability), environmental factors (e.g., resource use, ecological impact), or characteristics of food production, distribution, and access systems (e.g., supply chain efficiency, equity of access).

## 5 Conclusion

This study has demonstrated that the prevalence of inadequate apparent intake of available protein, lysine, and tryptophan is substantially higher compared to their apparent inadequate total protein, lysine and tryptophan, underscoring the importance of protein quality in nutrition security. The inadequacy of these nutrients was significantly associated with child nutritional status, with stunted children exhibiting higher prevalence than their non-stunted counterparts. We have also identified regional and residential (rural vs. urban) disparities in nutrient inadequacy, with the Central region and rural areas experiencing the highest burden. Tryptophan inadequacy was associated with household headship, with higher prevalence observed in female-headed households than in male-headed households.

## 6 Policy and future research directions

Target policy actions such as promoting rearing and consumption of small stocks alongside production of quality protein maize is required to address the protein and essential amino acids inadequacy among children. Further, the integration of gender-responsive nutrition education to incorporate dietary diversity into poverty reduction programmes particularly in the central region and rural areas are required to address protein and lysine inadequacy and reduce stunting. Future studies should also assess the effectiveness of income-focused interventions directed at female heads of household, such as employment support and expanded access to credit on household food and nutrition security and child nutritional status. Moreover, future studies may also examine sustainability of protein, lysine and tryptophan in relation to social and economic dimensions (e.g., household income, food affordability), environmental factors (e.g., resource use, ecological impact), or characteristics of food production, distribution, and access systems (e.g., supply chain efficiency, equity of access).

## Data Availability

Publicly available datasets were analyzed in this study. This data can be found here: https://microdata.worldbank.org/index.php/catalog/3818.
